# Spatial structure and nest demography reveal the influence of competition, parasitism and habitat quality on slavemaking ants and their hosts

**DOI:** 10.1186/1472-6785-11-9

**Published:** 2011-03-28

**Authors:** Inon Scharf, Birgit Fischer-Blass, Susanne Foitzik

**Affiliations:** 1 Department of Biology II, Ludwig Maximilian University of Munich, Germany; 2 Institute of Zoology, Johannes Gutenberg University of Mainz, Germany

## Abstract

**Background:**

Natural communities are structured by intra-guild competition, predation or parasitism and the abiotic environment. We studied the relative importance of these factors in two host-social parasite ecosystems in three ant communities in Europe (Bavaria) and North America (New York, West Virginia). We tested how these factors affect colony demography, life-history and the spatial pattern of colonies, using a large sample size of more than 1000 colonies. The strength of competition was measured by the distance to the nearest competitor. Distance to the closest social parasite colony was used as a measure of parasitism risk. Nest sites (i.e., sticks or acorns) are limited in these forest ecosystems and we therefore included nest site quality as an abiotic factor in the analysis. In contrast to previous studies based on local densities, we focus here on the positioning and spatial patterns and we use models to compare our predictions to random expectations.

**Results:**

Colony demography was universally affected by the size of the nest site with larger and more productive colonies residing in larger nest sites of higher quality. Distance to the nearest competitor negatively influenced host demography and brood production in the Bavarian community, pointing to an important role of competition, while social parasitism was less influential in this community. The New York community was characterized by the highest habitat variability, and productive colonies were clustered in sites of higher quality. Colonies were clumped on finer spatial scales, when we considered only the nearest neighbors, but more regularly distributed on coarser scales. The analysis of spatial positioning within plots often produced different results compared to those based on colony densities. For example, while host and slavemaker densities are often positively correlated, slavemakers do not nest closer to potential host colonies than expected by random.

**Conclusions:**

The three communities are differently affected by biotic and abiotic factors. Some of the differences can be attributed to habitat differences and some to differences between the two slavemaking-host ecosystems. The strong effect of competition in the Bavarian community points to the scarcity of resources in this uniform habitat compared to the other more diverse sites. The decrease in colony aggregation with scale indicates fine-scale resource hotspots: colonies are locally aggregated in small groups. Our study demonstrates that species relationships vary across scales and spatial patterns can provide important insights into species interactions. These results could not have been obtained with analyses based on local densities alone. Previous studies focused on social parasitism and its effect on host colonies. The broader approach taken here, considering several possible factors affecting colony demography and not testing each one in isolation, shows that competition and environmental variability can have a similar strong impact on demography and life-history of hosts. We conclude that the effects of parasites or predators should be studied in parallel to other ecological influences.

## Background

Enemy-victim interactions are not only affected by local densities in a certain habitat, but also by the movement of individuals and their location [e.g. [1-3]]. Animals clearly interact more often with neighbors than with more distant individuals. Nevertheless, many predator-prey models are spatially-implicit [sensu [4]]. The analysis of positioning or spatial pattern can lead to a better understanding of species interactions. Studies based on species densities alone might miss important local interactions, as if they analyze these interactions on a too coarse scale [5]. Predators and parasites reduce the fitness of their victims and they affect many behavioral and life-history traits [e.g. [6,7]]. For example, prey/host individuals avoid risky habitats, decrease foraging in general, reach smaller body size or require a longer development time [8]. Such behavioral and life-history responses are costly.

Behavioral and life-history changes are context-dependent and operate in parallel with other factors, such as competition and resource availability. For instance, predation risk is often ignored if the prey risks starvation [e.g. [9]]. Competition, ever present in nature, can force individuals to increase foraging effort [e.g., by facing interference from other competitors or depletion of food resources [10]]. Animals, therefore, face conflicting demands. For example, coping with predation might require a change in habitat usage, which is different from its usage under high parasitism risk [11]. The spatial pattern can also be affected in opposite ways. Competition for space or food often results in a regular spatial pattern, as individuals maximize the distance to other competitors. Predation risk, however, frequently leads to a clumped spatial distribution of prey, to dilute the risk [12,13]. Therefore, studies combining predation/parasitism with other important biotic and abiotic factors can better and more reliably estimate the relative importance of predation/parasitism for prey populations.

We study here the effect of parasitism risk combined with competition and an abiotic limiting factor on host ant colonies in two ecosystems of slavemaking-host ants. Slavemaking ants are obligate social parasites that depend on enslaved host workers recruited during recurrent slave-raids from host nests [14-16]. Slavemaking ant workers are incapable of foraging and taking care of their brood. They specialize on invading host nests and stealing their brood, killing host workers and occasionally the queens. Stolen host brood is raised in the slavemaker nest, and emerging host workers serve as slaves [14-16]. Removing/adding social parasites induce changes in host life-history and densities, which are species and population-specific [17,18]. Two general responses were evident in plots populated by slavemaking ants. In one population, host colonies under parasite pressure were more related, contained fewer queens and workers and had a lower brood production. In another population, host densities were reduced with a milder impact on social structure and life-history [18]. Slavemaking ants fit the definition of 'micro-predators'. They attack more than one host colony, unlike typical parasites (which attack one host), and exploited host individuals are not necessarily killed following attacks, unlike typical predators (which kill their prey) [19]. Slavemaking ants resemble in some aspects parasites, and in others predators (e.g., raiding dynamic). Comparisons to parasite-host/predator-prey interactions are imperfect, but based on the rich literature of both interactions, they allow drawing testable predictions.

The life-history responses of host ants to slavemaking ants emerged from comparisons between plots rich and poor with slavemaking ants, and from experimental manipulations during which slavemaker nests were removed/added. Yet, spatial pattern were not reported and within-plot differences among colonies are not accounted for [17,18,20,21]. We define "fine scale" as referring to within-plot spatial heterogeneity (i.e., colonies interact only with neighbors), and "coarse scale" as referring only to colony densities within the plots (i.e., neighbors are not expected to interact more frequently). Previous studies used only "coarse scale" (as defined here) in their analysis, and also did not inspect the relative importance of different explanatory variables [e.g. [17,18,20,21]]. Slavemaking ants can strongly influence their hosts, but the relative importance of this selective force would only be revealed when placed in a larger context, that is when statistical analyses include additional explanatory variables.

We use three large datasets of host and slavemaking ant colonies in three communities - New York and West Virginia (hereafter NY and WV), USA, and Bavaria, Germany - which include detailed data on colony demography (e.g., number of workers and social structure) and the size of the nest site. We study how local competition, parasitism risk and limitation for nest sites affect spatial pattern, colony demography and brood production of host colonies. We also test for effects of host density and spatial pattern on the slavemaker ants' demography. We use the same analytical tools to compare these communities (composed of different species) in order to reveal the relative importance of different factors in shaping these species interactions. While habitats and ant communities differ to a certain extent, the species interactions are comparable (see Methods for a detailed description). Comparing related systems is important for generalization and understanding how natural selection leads to similar solutions when the environment is similar [e.g., a comparison between the foraging behavior of desert rodents in two continents [22]].

We have two main objectives: (1) comparing the relative importance of different selective factors in three communities of ants; (2) better understanding the importance of scale when studying species interactions. Our working hypothesis is that interactions are space-dependent - colonies interact more frequently with immediate neighbors. We predict therefore that host colonies residing close to a slave-making colony or in a locally denser microhabitat should suffer from parasitism or competition more than host colonies located further away. This difference should be reflected in life-history traits (a negative correlation between competition or parasitism intensity and colony size, brood production and per-capita productivity). Alternatively, if there is strong local variation in resource availability, colonies in denser high-quality areas could be more successful than colonies in poorer sites of low density. In this case, a negative correlation between distance to the nearest neighbor and life-history traits could be found. Similar to other systems, we expect the colony spatial pattern to be regular on a fine scale and clumped on a coarser scale [23,24]. This shift results from different limiting factors on different scales (finer scales - competition; coarser scales - environmental heterogeneity). We also expect that as colony densities increase, the spatial pattern should be more regular, as colonies try maximizing the distance to the nearest competitor.

We hypothesize that the relative influence of parasitism risk, competition and nest site limitation would be system-dependent. We predict that nest site limitation would be a universal factor, more dominant than other factors. High quality nest sites are a limiting resource in such ecosystems, and a positive correlation between colony size, brood production and nest site size should be evident, due to competition for nest sites. Competition should be important in Bavaria, which is already known as a poor relatively sparsely-populated habitat. Parasite pressure should be higher in WV and NY, and especially in the latter, as slavemaking ants in NY inflict larger damage to host colonies compared to other habitats [18]. Slavemaking nests having closer host neighbors should reside in areas with a higher host density, and thus possess larger more productive colonies. Alternatively, virulent slavemaking ants may have only few host neighbors, because they already destroyed all nests nearby.

## Methods

### Study ecosystems and sites

We used a dataset of host and slavemaker ant locations and demography in NY (Edmund Nils Huyck preserve; 42°32'N, 74°9'W; 500 m a.s.l.) and WV (Watoga state park; 38°6'N, 80°8'W; 850 m a.s.l.) [[18], unpublished data]. The main host ant species, *Temnothorax longispinosus*, is parasitized by the slavemaking ant *Protomognathus americanus *and constituted between ~85% (NY) and ~95% (WV) of all collected ant nests. Other host species collected were *T. curvispinosus *in WV and *T. ambiguus *in NY, and colonies of those species were removed from later analysis. The datasets include 18 and 21 sampled plots in NY and WV of 25 m^2 ^each, in which all colonies were collected and censused (May 2001). We have data on each colony location, number of queens, workers and larvae. *T. longispinosus *is a facultatively polydomous species (i.e., colonies may occupy more than a single nest) [25]. The identification of colony boundaries requires behavioral and genetic analyses, and is not trivial [26]. For our large sample size, this work-intensive procedure is not feasible. We thus follow earlier studies on *Temnothorax *ants, suggesting that selection operates on the nest level in this species [27,28]. Since colonies were collected before summer, there was still no production of sexuals, but only larvae. Colony densities per m^2 ^in the field were higher in NY 0.904 ± 0.362 (mean ± 1 S.D.) than in WV (0.604 ± 0.377; Mann Whitney U test: U = 227.0, P = 0.013, N = 18,21). In further analysis (spatial pattern and demography) we refer to the *P. americanus *as the slavemaker and *T. longispinosus *as the host. Total number of host and slavemaker colonies was 407 and 33 (NY) and 317 and 35 (WV).

In addition, we used a dataset of ant locations and demography from a community in Bavaria, Germany (~8 km east of Abensberg; 48°49'N, 11°58'E; 400 m a.s.l.) [[29], unpublished data]. The European ecosystem is slightly more complex, involving two host species at about the same rate, the larger *Leptothorax acervorum *and the smaller *Leptothorax muscorum*, parasitized by the slavemaker *Harpagoxenus sublaevis*. A third species in that community, *Temnothorax crassispinus*, does not serve as a host for the slavemaking ant, but is a potential competitor for the two host species. We therefore include it in the spatial pattern, but do not analyze its demography. The dataset includes 20 plots of 100 m^2 ^each, in which all colonies were collected and censused in July-August 2001. We possess information on colony location, number of queens, workers, production of sexuals, pupae and larvae. Colony densities in the field are 0.158 ± 0.056 (mean ± 1 S.D.) and are only 18-30% of the nest densities in the North American ecosystem. Total number of host (of the two species) and slavemaker colonies was 315 and 31 (we had exact coordinates in the collection site of only 28).

**Figure 1:**
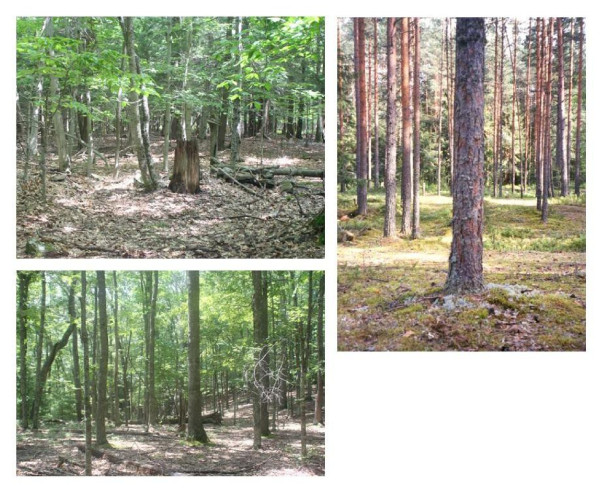
**Photos of the collection areas (clockwise: New York, Bavaria and West Virginia)**. The two American forests exhibit higher tree species diversity than the Bavarian monoculture pine forest.

The three collection areas belong to larger forests. The collection sites in the USA are a part of nature preserves, undisturbed at least since ~1930 (Huyck preserve website: http://www.huyckpreserve.org/index.htm; Watoga park website: http://www.pocahontascountywv.com/watoga_state_park.aspx). Both are characterized by a diversity of trees, dominated by different oak species (*Quercus *spp.) and hickory (*Carya *spp.), including also maple trees (*Acer *spp.) (Figure 1). The forest near Abensberg, Bavaria, is a monoculture of a single pine tree, *Pinus sylvestris *(Figure 1). The North American ant species nest in acorns, nuts or small wood sticks in the leaf litter layer of deciduous forests [20], while the European ants inhabit pine forests and invariably nest in pine sticks and logs on the forest floor. We documented in all datasets the size of the nest site. As a measure of nest site size, we refer to the diameter of the organic structure (e.g., stick, acorn, etc.) in which the colony resides. For long sticks we measured the diameter at the cavity of the colony.

The three studied ecosystems and ant species are ecologically similar, but also show several dissimilarities. All hosts and social parasites are small cavity-dwelling ants of temperate forests, residing in woody structures [e.g. [25,30-32]]; colonies relocate frequently due to the decomposition of their ephemeral nest sites [31]; they are phylogenetically related [33]; the ecological factors shaping the host ants' natural history are similar [e.g., competition, risk of parasitism by slavemakers, and environmental effects [18,25,30,34]]; and their colony size, social structure and foraging behavior are alike [e.g. [35-37]]. The main dissimilarities are the higher complexity of one ecosystem in which the slavemaking ant parasitizes two (and not one) host species at similar rates, resulting in different selective pressures; the forest tree species composition; and the polydomous nature of one host species (i.e., a colony occupying more than one nest) compared to all other monodomous species.

### Statistical analysis

#### 1. What is the spatial pattern of host colonies?

We applied all statistical analyses separately to the three communities, unless else mentioned. We estimated the spatial pattern by designing a null model, similar to previous studies [23]. Null models keep some of the data elements fixed while randomizing the rest, creating a pattern that is expected in the absence of any driving mechanism [38]. We randomly redistributed for each plot the same number of colonies originally residing in the plot 1000 times and estimated the expected distances of each colony to the 1^st^-6^th ^nearest neighbor. We then divided the real mean distances to the k^th ^nearest neighbor of all colonies in a plot by the grand mean of 1000 expected mean distances to the k^th ^nearest neighbor, derived from random distribution. We received six values for each plot in each habitat, representing the 1^st^-6^th ^nearest neighbor indices (hereafter NNI). Values larger and smaller than one indicate regular and clumped spatial patterns, respectively. We used the percentile bootstrap [39] on all plots within each community to estimate the 95% confidence limit intervals for the NNIs. See Additional File 1 for flow charts of all null models. In order to test for an effect of host and slavemaker densities within plots on the spatial pattern, we used linear regressions, with host or slavemaker densities and NNIs as explanatory and dependent variables respectively. Simulations and statistical analyses were performed in MATLAB v.6.5 (Mathworks, Natick, USA) and SYSTAT v.11 (SYSTAT Software, San Jose, USA).

#### 2. Are the slavemaking colonies correlated in space with host ant colonies?

We refer to this question on coarser (among plots, no reference to space) and finer (within plots, relating to distances to nearest neighbors) scales. We first correlated the slavemaker and host colony abundance in each plot, for each community separately, using three linear regressions (i.e., host densities explaining slavemaker densities). We used a similar null model to analyze if the slavemaking colonies are closer to host colonies than expected (i.e., analysis on a finer scale). We randomized the location of each slavemaking colony for 1000 times and recalculated the distance to its nearest host colony within each plot. We calculated 95% limit intervals for the obtained distribution of distances. When the true distance to the nearest host falls within these limits, the slavemaker is neither closer to nor more distant from the hosts than randomly expected. We repeat it for the 2^nd^-6^th ^nearest neighbors, to test for a similar relationship with more distant neighbor.

#### 3. Are both host species positively auto-correlated in space?

This question was relevant only to the Bavarian dataset including two potential host species, and we analyzed the data on coarser and finer scales. We correlated the densities of the two host species in all plots. Species abundance can be either positively correlated pointing to a favorable microhabitat, or negatively correlated, owing to inter-specific competition. Species auto-correlation on a finer scale was tested by a similar null model. We calculated in each plot for each species the proportion of colonies having nearest neighbors of the same species. Next, we reshuffled the species types (but not locations) for 1000 times and recalculated the proportion of colonies having nearest neighbor of the same species. We then subtracted the observed proportions for each plot from the mean proportion expected by chance, calculated by averaging the proportions in 1000 replications. We created 95% confidence limit intervals using the percentile bootstrap. If zero is included in the obtained distribution, species are not more correlated than randomly expected.

#### 4. Which factors affect host and slavemaker colony demography?

We present here a fine-scale analysis, i.e., a within-plot analysis referring to nearest neighbors and slavemaking colonies in contrast to simply comparing colony densities. We used general linear models (GLMs) to test for the effects of distance to nearest host competitor colony (nearest neighbor distance: NND) as a proxy of competition, distance to nearest slavemaking colony (nearest slavemaker distance: NSMD) as a proxy of parasitism risk, nest site size (NS) and all possible interactions on the number of workers, queens, immature stages (only larvae in NY and WV), males and new queen production (only in the Bavarian dataset), and per-capita productivity (immature stages and sexuals/number of workers). We performed the analyses separately for the three communities, and used plot as an additional explanatory variable. In NY and WV communities there was only one host species, while in Bavaria we analyzed both host species and use also species as an explanatory variable. We also documented if the nearest neighbor is the same host species or another species (NNSp). To disentangle between intra-specific and inter-specific competition we included it in the analysis. We tested for all possible interactions of species with other explanatory variables. NNSp interactions included only NNSp × NND. The competitor unparasitized species *T. crassispinus *was not included. We referred only to parasitized plots, because NSMD was always part of the analysis. All variables were log transformed, since they were not normally distributed.

We used a model selection procedure to decide which interactions should be included in each model, including the Akaike information criterion corrected for small sample size (hereafter, AICc) [40]. We started each time with a saturated model and gradually removed the least significant interaction. We recorded the number of parameters (main effects, interactions, constant and error term) and the RSS (residual sum of squares) for the calculation of the AICc. We stopped the removal process when the model included only one explanatory variable. The best model was chosen according to the minimal AICc values [40]. The best model may include not significant factors or remove significant factors, depending on the RSS, sample size and number of parameters in the model.

We were interested whether the same results were evident when using a coarser spatial scale - plot densities instead of NNDs, as another proxy of competition. For that purpose we tested using a GLM the effect of plot density while accounting for nest site size and plot (adding nest size and plot number as explanatory variables) on number of workers and total production in the Bavarian dataset. We used only this dataset, because the strongest effect of competition was evident there on a fine scale.

Slavemaker demography was analyzed using model selection. Size of the nest site, distance to nearest host and the two-way interaction were treated as explanatory variables. Slavemaker workers and number of slaves were referred to as the dependent variables. We removed the interaction and each of the two main effects and chose the best model according to AICc. Due to the small sample size of slavemaker colonies, we pooled over plots.

#### 5. Do ant communities differ in habitat heterogeneity?

The previous analyses can provide indications on how variable resources are distributed in space in the different habitats. For example, if ant colonies are more productive in dense sites, we would conclude that competition is less important and resource availability varies in space. To investigate habitat heterogeneity in more detail, we calculated for each community the coefficient of variation (CV) of the abiotic and biotic community characteristics within each plot (CV of worker number, total production and size of the nest site). These values represent heterogeneity within plots. We used three one-way ANOVA with community (Bavaria, NY and WV) as the explanatory variable and each of the above as dependent variables.

## Results

### 1. What is the spatial pattern of host colonies?

**Figure 2:**
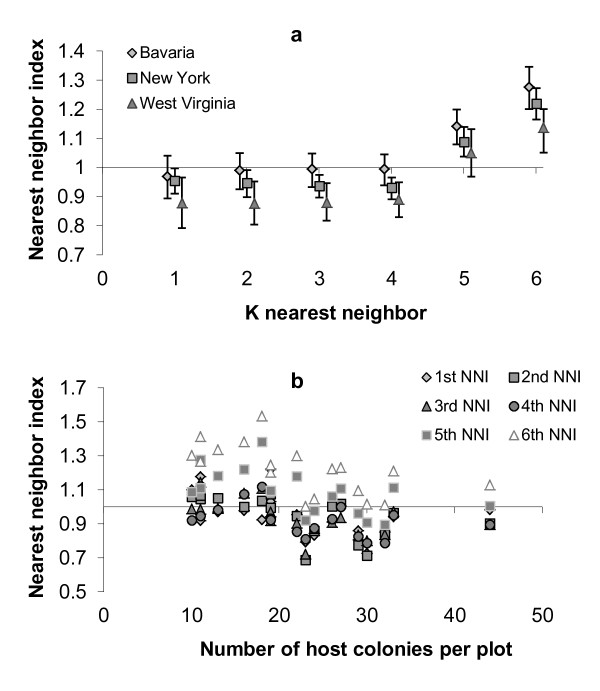
**(a) 1^st ^to 6^th ^nearest neighbor indices for the three populations (means ± 95% confidence limit intervals)**. Confidence limit intervals below and above 1 suggest clumped or regular patterns, respectively. 1^st ^to 4^th ^NNIs point to a more clumped spatial pattern while the 5^th ^to 6^th ^NNIs suggest a more regular one. (b) The negative correlation of host colony densities in each plot in the NY population with the spatial pattern (NNI), for the 1^st ^to 6^th ^nearest neighbor indices. Nearest neighbor distances decrease as density increases, but the NNI is already corrected for host density.

Host colonies were slightly clumped on a finer scale when considering the immediate neighbors (the 1^st^-4^th ^nearest neighbors; Figure 2a). This trend was the strongest in WV and the weakest in Bavaria, where the spatial pattern was random (the value of 1 falls within the 95% confidence limit intervals). On a coarser scale colonies became over-dispersed in all communities (the 5^th^-6^th ^nearest neighbors; Figure 2a). Linear regressions found no significant correlations between either host or slavemaker densities and nearest neighbor indices either in Bavaria or WV (all P > 0.05). However, negative correlations were evident in NY for most NNIs: colonies aggregate more with increasing density of both hosts and slavemakers (see Additional File 2 for the statistical significance). The interaction term, host × slavemaker densities, was not significant (all P > 0.05).

### 2. Are the slavemaking colonies correlated in space with host ant colonies?

On a coarser scale, we tested for a correlation between host and slavemaker densities in each plot. We could not find a significant association between host and slavemaker density either in NY (F_1,16 _= 0.52, P = 0.48, R^2 ^< 0.001) or Bavaria (F_1,18 _= 0.65, P = 0.43, R^2 ^< 0.001), but a positive correlation was found in WV (F_1,19 _= 14.79, P = 0.001, R^2 ^= 0.408). On a finer scale, the null model suggested that slavemaker colonies are usually not closer than expected to host colonies, independent of the NNI used (1^st ^to 6^th^). In Bavaria 23-25 of 28 slavemaker colonies (82.1-89.3%) were inside the randomly expected range of distances from host colonies for all 1^st ^to 6^th ^nearest neighbor hosts. Similar proportions could be shown in NY (84.9-90.9%, 28-30/33) and WV (90.9-94.3%, 30-33/35).

### 3. Are both host species positively auto-correlated in space?

This question was relevant only to the Bavarian dataset. On a coarser scale, we tested for an association between the two species abundances in every plot using a Pearson correlation. Host densities were significantly and negatively correlated (Bartlett χ^2 ^statistic = 5.64, df = 1, P = 0.018). However, on a finer scale and according to the null model, both species were not more auto-correlated than expected (confidence limit intervals of [-0.131,0.007] and [-0.137,0.004] for *L. acervorum *and *L. muscorum*, respectively). This suggests that the probability for each host species to have a nearest neighbor of the same or other species is similar, given the abundance of both species within each plot. See Additional File 3 for further analysis of the Bavarian community.

### 4. Which factors affect host and slavemaker colony demography?

**Figure 3:**
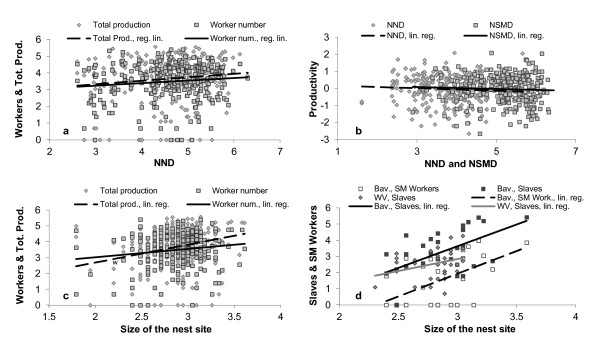
**The influence of competition and nest site size on various life-history variables.** (a) The correlation of distance to nearest conspecific colony with number of workers (squares, continuous line) and total production (diamonds, dashed line) in the Bavarian community. (b) The correlation of distance to nearest conspecific (diamonds, dashed line) and slavemaking colony (squares, continuous line) with colony per-capita productivity in the NY community. (c) The correlation of size of the nest site with workers (squares, continuous line) and total production (diamonds, dashed line) in the Bavarian community. (d) The correlation of nest site size with number of slaves (black squares, continuous line) and slavemaking workers (white squares, dashed line) in the Bavarian community, and of nest site size with number of slaves (grey diamonds, grey line) in the WV community. All variables are log transformed.

The models selected according to AICc are presented in Table 1. In several cases the difference between the best and second best models was very small (difference in AICc values < 0.7; Table 1). The three-way interactions, host species (in the Bavarian community) as well as the species identity of the nearest neighbor (NNSp) or its interaction with the distance to the nearest neighbor (NNSp × NND) were never included in the best model and are therefore absent from Table 1. In general, the Bavarian community was negatively affected by density or intra-guild competition. Worker number, total production and per-capita productivity increase with distance to the nearest neighbor (Figure 3a). In the NY community there was a negative correlation of per-capita productivity with NND and NSMD: the per-capita productivity of host colonies is higher in close vicinity to conspecific and slavemaker colonies (Figure 3b). Finally, WV community was affected mostly by size of the nest site, which had an effect on the two other communities as well (Figure 3c). In the Bavarian community, number of workers and total production were not affected by plot density in contrast to the finer-scale analysis presented in Table 1 (number of workers: F_1,276 _= 0.78, P = 0.38; total production: F_1,286 _= 0.14, P = 0.71).

**Table 1:** Factors affecting host demography and life-history.

	Plot	NND	NSMD	**Nest Diam**.	Inter. (NND × NSMD)
Bavaria	Slavemker: *H. sublaevis*		Hosts: *L. acervorum, L. muscorum*		

Workers	F_16,244 _= 1.41 P = 0.139	F_1,244 _= 3.89 P = 0.050 (+)	-	F_1,244 _= 9.39 P = 0.002 (+)	-
Queens	F_16,255 _= 3.43 P < 0.001	-	-	-	-
Total Prod.	F_16,252 _= 1.09 P = 0.363	F_1,252 _= 7.15 P = 0.008 (+)	-	F_1,252 _= 17.77 P < 0.001 (+)	-
Product.	F_16,245 _= 1.62 P = 0.065	^† ^F_1,245 _= 2.92 P = 0.089 (+)	-	-	-
Queen Prod.	F_16,253 _= 2.74 P < 0.001	-	-	^§ ^F_1,253 _= 5.18 P = 0.024 (+)	-
Male Prod.	F_16,253 _= 2.25 P = 0.005	-	-	F_1,253 _= 7.48 P = 0.007 (+)	-

NY	Slavemaker: *P. americanus*		Host: *T. longispinosus*		

Workers	F_14,295 _= 2.32 P = 0.005	-	-	^¥ ^F_1,295 _= 5.17 P = 0.024 (+)	-
Queens	F_14,299 _= 2.17 P = 0.009	-	-	-	-
Larvae	F_14,296 _= 2.01 P = 0.017	-	-	-	-
Product.	F_14,275 _= 5.79 P < 0.001	F_1,275 _= 6.27 P = 0.013 (-)	F_1,275 _= 5.96 P = 0.015 (-)	-	F_1,275 _= 6.38 P = 0.012

WV	Slavemaker: *P. americanus*		Host: *T. longispinosus*		

Workers	F_12,212 _= 2.33 P = 0.008	-	-	^£ ^F_1,212 _= 3.08 P = 0.081 (+)	-
Queens	F_12,216 _= 0.63 P = 0.818	^‡ ^F_1,216 _= 3.90 P = 0.050 (+)	-	-	-
Larvae	F_12,210 _= 2.39 P = 0.007	-	-	F_1,210 _= 10.32 P = 0.002 (+)	-
Product.	F_12,210 _= 1.70 P = 0.068	-	-	F_1,210 _= 6.12 P = 0.014 (+)	-

The best model was selected using model selection procedure (AICc). NND stands for nearest neighbor distance, NSMD for nearest slavemaker distance, Prod. for production, and Product. for (per-capita) productivity. Three-way interactions and species as well as the species identity of the nearest neighbor (the latter two relevant only to the Bavarian dataset) were never included. The only two-way interaction included is NND × NSMD. (+) and (-) indicate a positive or negative correlation respectively.

^† ^The best model did not include NND, but the second best, which had a close AICc value (difference of 0.71), included it.

^‡ ^The best model included NND, but the second best, which had a close AICc value (difference of 0.69), did not.

^§, ¥ ^The best model included NS, but the second best, which had a close AICc value (difference of 0.32 (§) and 0.29 (¥)), did not.

^£ ^The best model did not include NS, but the second best, which had a close AICc value (difference of 0.66), included it.

Slavemaker colonies were affected by the size of their nest site (Figure 3d) with little effect of distance to nearest host. The Bavarian slavemaker population was only affected by the size of the nest site (best model included only nest site size: Workers: F_1,25 _= 10.72, P = 0.003, R^2 ^= 0.272; Slaves: F_1,25 _= 9.59, P = 0.005, R^2 ^= 0.248). The NY population was influenced by none of the explanatory variables: no model explained well the dependent variable (Workers: F_1,30 _= 2.26 P = 0.14, R^2 ^= 0.039; Slaves: F_1,31 _= 1.48, P = 0.23, R^2 ^= 0.015). WV slavemaker population was affected by the distance to nearest host (Workers: F_1,33 _= 4.61, P = 0.039, R^2 ^= 0.096) and nest site size (Slaves: F_1,31 _= 3.61, P = 0.067, R^2 ^= 0.075). Significant correlations were always positive (Figure 3d).

### 5. Do ant communities differ in habitat heterogeneity?

**Figure 4:**
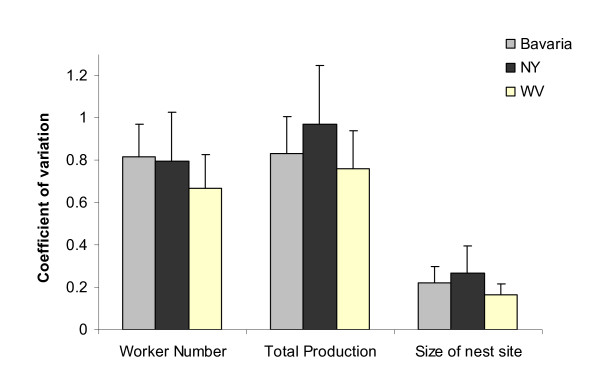
**Coefficient of variation (CV) of three important community variables (worker number or colony size, total production and nest site size) within plots**. Means ± 1 SD are presented. Sample sizes are 20 (Bavaria), 18 (NY) and 19 (WV).

Overall, NY showed a higher heterogeneity within plots compared to the two other communities, and especially WV (Figure 4). CV of worker number within plots differed among communities (F_2,54 _= 3.78, P = 0.029; LSD post-hoc test: Bavaria and NY > WV), which was higher in Bavaria and NY. CV of total production differed as well among communities and was the highest in NY, followed by Bavaria and WV (F_2,54 _= 4.80, P = 0.012; LSD post-hoc test: NY > WV). CV of the size of the nest site showed a similar pattern to the CV of total production (F_2,54 _= 6.61, P = 0.003; LSD post-hoc test: Bavaria and NY > WV).

## Discussion

We used three datasets to investigate different aspects of competition in ants (distance to the nearest neighbor, spatial pattern and size of the nest site), relationships of host ants with their slavemaking parasites (distance to the nearest slavemaker) and an abiotic limited resource (nest sites). This combination represents selective forces which affect demography and life-history differently in each habitat. Competition was apparent in Bavaria, while habitat heterogeneity dominated in New York. Suitable nest sites were the only universal limiting factor in these ecosystems. Analysis on finer scale (relating to intra-plot spatial positioning of colonies and to the nearest neighbors) and coarser scales (referring only to plot densities) often provided different results (Table 2). Previous studies focused on social parasitism and its effect on host colonies [e.g. [17,18,29]]. We use a broader approach, referring in parallel to competition and abiotic factors. Such factors have a similar strong impact on demography and life-history of hosts.

**Table 2:** Comparison of different aspects on a finer and coarser scale.

	Spatial scales	
	Fine	Coarse
Spatial pattern	Clumped	Regular
Do slavemaker colonies reside next to host colonies?	No spatial correlation *	Yes, but only in WV
Are host species correlated in space (Bav.)?	No correlation	Yes, negatively correlated
Do slavemaker colonies prefer one of the host species (Bav.)? **	No correlation	No correlation
Does competition/density affect life-history of host colonies (Bav.)?	Yes ***	No

* No spatial correlation for the 1^st ^to 6^th ^nearest host.

** See Additional File 3 for this analysis.

*** When testing for the effect of NND vs. the number of colonies in each plot on number of workers and total production.

Host and slavemaking colonies move for several times during the season due to the decomposition of their nest sites [e.g. [25,31]]. Nevertheless, using the distances to the nearest competitor or slavemaking colony is a suitable proxy of competition or parasitism risk. Available nest sites dictate the colonies' locations, but nest sites are probably clustered in specific locations in the experimental plots. Therefore, even after relocating, colonies remain close to their previous location. There is no data on relocation distances in these species, but only on proportion of relocating colonies throughout the season [21]. The maximal distance in a related species is 0.5 m (*T. nylanderi*; S. Foitzik, personal observations). Therefore and owing to the inefficient recruitment of the ants, the relocation distances are probably short, so competition, parasitism risk and the spatial pattern should not change much following relocations. A more thorough experiment following individual colonies would be helpful. However, it is impossible to obtain data on the colony locations without destroying their nests and initiating emergency relocations.

### Interactions on fine vs. coarse scales

Competitive and parasitic interactions depend on the scale of measurement. On a finer scale, slavemaking colonies are not closer to their host colonies than randomly expected, a pattern found to be valid on a coarser scale [WV in the current study; NY in a previous study [20]]. The maximal distance of slave-raids in natural populations is unclear, but polydomous nest parts of a single parasite colony were found between 0.26 and 5.56 m apart [41,42]. Specifically in this case a too fine scale may be of less importance, because slavemakers can reach all parts of the study plots. Slave-raids could also lead to a negative spatial correlation between host and slavemaker colonies. This was also not the case. Even if slavemakers clear-out their immediate surroundings from host colonies, these empty nest sites can be re-populated fast by new colonies [41] owing to strong competition for suitable nest sites [30].

In Bavaria, competition had a strong effect on various life-history traits on a finer scale, and host species did not show a spatial positive correlation. In comparison, no evidence for competition was evident on a coarse scale, but densities of host species were negatively correlated. The spatial pattern differed among scales as well in all communities. Spatial correlations on a finer scale were perhaps weaker because ant colonies sometimes relocate their nests due to the decomposition of the nest site [e.g. [30,31]]. These differences stress that more than one scale should be used for analysis and that the proper spatial scale for each question should be carefully determined and justified.

### The spatial pattern of host ants

The spatial pattern in all communities is relatively clumped on a finer scale relating to the immediate neighbors, but regular on a coarser scale, referring to more distant neighbors. The small clusters observed suggest that there are several 'hotspots' within plots, each can sustain several colonies. Such 'hotspots' can result from clumped spatial pattern of available nest sites, polydomy or satellite nests, which may induce patchiness in ant communities [43]. On a larger scale, other factors are probably more relevant for spatial patterns, such as the habitat structure (e.g., locations of trees). Studies of ant spatial pattern usually found a regular spatial pattern of colonies [44,45]. This was not the case in this study, possibly because the studied ants rely on specific nest sites, so that part of their spatial pattern could be explained by the spatial pattern of these nest sites on the forest floor.

Aggregation was mostly evident in NY and WV. The spatial patterns in NY on all scales (1^st^-6^th ^NNI) became more clumped as slavemaker densities increased, supporting the concept of aggregating as an anti-predator response. In a previous experiment, the NY host colonies responded to the presence of slavemaker by changes in life-history and not by reduced density, as was the case in WV [18]. Changes in spatial pattern may be another strategy to cope with social parasite presence, in addition to modifications in life-history. Such responses may enhance host colony survival (slavemaking ants reduce survival of hosts, mainly in WV). In other words, host colonies in NY are possibly more flexible in responding to the slavemaker by changing both some life-history traits and spatial pattern. However, the spatial pattern was more clumped with an increase in host densities as well, making it difficult to differentiate between a causative or a correlative relationship between slavemaker densities and the spatial pattern. When competition is strong, the spatial pattern is expected to be regular, as animals aim at maximizing distances to nearest-neighbors [13,24,45]. In accord, the Bavarian community showed the most regular spatial pattern, on all k NNI used (Figure 2a). This fits well the negative general impact of competition found in this community.

### Comparison of the dominant selective factors in each community

The positive correlation between the size of the nest site and different life-history traits was the most dominant factor in all communities for both hosts and slavemakers. Previous studies have demonstrated a positive correlation between the size of the nest site and the number of queens or workers in cavity-dwelling ants [25,30] and other ant species [e.g. [46]]. Nest sites also affect the per-capita productivity. Per-capita productivity may be influenced either directly or indirectly, since more populous colonies reside in larger nests, and such colonies may be more productive per worker. The latter is not the case as the number of workers is either negatively or not correlated with per-capita productivity (a negative correlation in WV: F_1,305 _= 4.34, P = 0.038, R^2 ^= 0.011; no correlation in Bavaria: F_1,302 _= 1.45, P = 0.23, R^2 ^= 0.001; not relevant for NY, because the size of the nest site was not correlated there with per-capita productivity). This finding indicates that residing in high quality nest sites per se raises colony per-capita productivity. Nest sites may restrict colony size, or larger colonies may more successfully compete for larger better nests. When potential nest sites are artificially augmented they are rapidly populated changing colony densities, sex ratios and sexual production [34]. Ants in crowded nests can have higher metabolic rates with negative effects on per-capita productivity and fitness [47]. We support this observation, by showing a positive correlation between nest site size and different measures of colony size and fitness.

The NY community was affected by habitat heterogeneity. Colonies were clustered in high-quality habitats as indicated by the negative correlations between per-capita productivity and distance to the nearest competitor. Heterogeneity was not measured directly, but the higher variance within plots in NY in colony size, nest size and total production point in this direction (Figure 4). It also fits to on-going research demonstrating that NY colonies in denser areas are more productive (Modlmeier and Foitzik, in review). The WV community was influenced only by the size of the nest site, with no significant effect of competition or parasitism risk. These findings support a previous comparative study, showing a stronger demographic effect of slavemaker colonies on hosts in NY than in WV [18]. Some microhabitats inside the plots in NY were probably favorable for ants, resulting in dense clusters, possibly related to the occurrence of suitable nest sites which are not too degraded. Per-capita productivity decreased as moving out of these clusters.

In Bavaria, as ant colonies are closer, they are smaller, produce less brood and have lower per-capita productivity, all demonstrating the negative effects of competition. In this case, the finer scale analysis with a reference to nearest-neighbor distances produced stronger results. Ryti and Case [45] showed the same positive correlation between distance to nearest-neighbor and colony size in species which are usually over-dispersed. Other studies found first a positive correlation between distance to nearest neighbor and alate production but later on failed to find a similar correlation in the same ecosystem (compare [48] with [49]). It is suggested that local high densities may induce competition only when resources are limited. We suggest the same: the Bavarian community was not as dense as the American ones, but competition there was more evident, probably owing to resource limitation. We could not detect any difference between intra- and inter-specific competition types in Bavaria, as the identity of nearest neighbor had no effect on any of the colony life-history variables.

The Bavarian community was sampled later in the season than the American ones. This difference may have resulted in a weaker footprint of parasitism risk in the USA, because slave-raids occur usually later in the season. The expectation, therefore, is for a stronger parasitism risk in Bavaria, since slave-raids were on-going at sampling time. We actually found the reverse. In addition, similar to predation risk, parasitism risk can result in direct effects (colonies are invaded and robbed) and indirect ones (colonies change life-history traits to better cope with that risk). The latter should not be affected by the different sampling time. Other basic differences among habitats and ecosystems probably have consequences for the results achieved. For example, the Bavarian species are monodomous while the American ones are polydomous. It is difficult to guess how competition, parasitism risk and limitation of nest sites will affect polydomy and vice versa. We nevertheless suggest that polydomy may increase if parasitism risk or competition are strong, as a way to spread the risk (similar to bet-hedging), and if large nest sites are rare.

The slavemaker demography was affected by the size of their nest site. We could not show other influential factors possibly since our sample size is much lower for the slavemakers compared to host colonies. Nevertheless, it also suggests that slavemaker colonies are less spatially dependent than host colonies. Predators in general have a larger habitat range and they interact with space on a larger scale than their prey [5]. Only in WV there was an effect of the distance to the nearest host on slavemaker colonies. It fits the strong effects slavemakers have on host colony abundance in this community [18]. In other words, since slavemaker ants decrease the abundance of host colonies, it is logical to assume that slavemaker colonies with closer neighbors either exploit their environment less efficiently or are perhaps just colonies in their infant stages. Slavemaker colonies are presumably more dependent on the exact locations of host colonies in WV compared to NY, simply owing to the lower abundance of host colonies in WV, where the slavemakers have to follow their hosts.

## Conclusions

We would like to stress the importance of null models for comparing existing patterns to random predictions, especially when regular statistics may fail. For instance, when testing whether the two host species in the Bavarian community are correlated in space, it was important to take into account the abundance of each host within each plot. Earlier analyses based on abundances of parasite and host colonies came to a different conclusion than the current study. A recent study [29] found a preference of the slavemaker *H. sublaevis *to one of its two hosts, while no preference from a spatial point of view was evident here. Furthermore, parasitism risk was found to be less important than previously suggested [29]. Hence, analyzing spatial patterns and locations of slavemakers and hosts improved our understanding of the ant slavemaker-host relationships and the impact of competition.

## Authors' contributions

IS wrote the manuscript, designed the null models and did the statistical analysis. BFB carried out the field and lab work in Bavaria. SF designed the study, helped in the field in Bavaria, conducted the field work in NY and WV and contributed to writing the manuscript. All authors read and approved the final manuscript.

## Supplementary Material

Additional file 1 **Flow charts of all null models**. Click here for file

Additional file 2 **Statistics for the relationship between host and slavemaker densities and spatial pattern in the NY community**. Click here for file

Additional file 3 **Further analysis of the Bavarian community**. Click here for file
