# Supplementary material for: Spatial structure and nest demography reveal the influence of competition, parasitism and habitat quality on slavemaking ants and their hosts
Source: BMC Ecol. 2011 Mar 28;11:9. doi: 10.1186/1472-6785-11-9 (PMC3078833; doi:10.1186/1472-6785-11-9)
Supplement: Additional file 1 — Flow charts of all null models. [file 1472-6785-11-9-S1.DOC]

**Additional file 1**

Title: Flow charts of the null models

Description: We present flow charts for the three models included in the paper. All null models are similarly designed and are based on randomizing colony locations or species identity in order to reach a random null expectation. The observed pattern is compared then to that expectation (Fig. A1): (**a**) null model testing for spatial pattern of host colonies; (**b**) null model testing for spatial correlation of slavemaking and host colonies on a finer scale; and (**c**) null model testing for spatial correlation of the two potential host species on a finer scale (in Bavaria). The null models are similarly designed, based on randomization of colony locations and comparisons of the observed patterns to the expected ones, assuming random spatial pattern.
