# Supplementary material for: Spatial structure and nest demography reveal the influence of competition, parasitism and habitat quality on slavemaking ants and their hosts
Source: BMC Ecol. 2011 Mar 28;11:9. doi: 10.1186/1472-6785-11-9 (PMC3078833; doi:10.1186/1472-6785-11-9)
Supplement: Additional file 2 — Statistics for the relationship between host and slavemaker densities and spatial pattern in the NY community. [file 1472-6785-11-9-S2.DOC]

**Additional File 2**

Title: The relationship between host and slavemaker densities and spatial pattern in the NY community

Description: Results of linear regression; host and slavemaker densities are treated as the explanatory variables and the spatial pattern (NNI) as the dependent variable. The interaction term, host × slavemaker densities, was not significant (all P > 0.05).

| k NNI | Model | | | Host | Slavemaker |
| --- | --- | --- | --- | --- | --- |
|  | F_2,15_ | P | R^2^ | P | P |
| 1^st^ NNI | 4.71 | **0.026** | 0.304 | 0.109 | **0.043** |
| 2^nd^ NNI | 15.43 | **<0.001** | 0.629 | **0.008** | **0.001** |
| 3^rd^ NNI | 12.35 | **0.001** | 0.572 | **0.012** | **0.003** |
| 4^th^ NNI | 6.24 | **0.011** | 0.381 | **0.044** | **0.034** |
| 5^th^ NNI | 7.36 | **0.006** | 0.428 | **0.019** | **0.037** |
| 6^th^ NNI | 11.89 | **0.001** | 0.562 | **0.006** | **0.009** |
