# Supplementary material for: Spatial structure and nest demography reveal the influence of competition, parasitism and habitat quality on slavemaking ants and their hosts
Source: BMC Ecol. 2011 Mar 28;11:9. doi: 10.1186/1472-6785-11-9 (PMC3078833; doi:10.1186/1472-6785-11-9)
Supplement: Additional file 3 — Further analysis of the Bavarian community. [file 1472-6785-11-9-S3.DOC]

**Additional File 3**

Title: Further analysis of the Bavarian community

Description: A spatial analysis using a simulation null model to understand whether the slavemaking colonies prefer one of the two host ant species.

Methods

This analysis is relevant only for the Bavarian dataset, which included two different host ants, *Leptothorax acervorum* and *L. muscorum*. We referred to this question on coarser and finer scales. First, we correlated the slavemaking colony abundance in each plot with each of the host species. We then used a null model, similar to those described in the paper, to test whether the nearest neighbor hosts belong more often than randomly expected to one of the two potential hosts. For each plot, we relocated a slavemaking colony 1000 times and determined which species is the nearest neighbor. We then calculated the proportion of times in which the slavemaker colony was closer to each host species by chance (expected pattern). We calculated how large the difference is between the observed and expected patterns. For example, if the parasite is closer to *L. acervorum* and the expected probability for such an event is 0.78, then the deviation from expectation is 0.22 (=1–0.78). When the deviations from expectations are large (i.e., above 0.5), there is a stronger preference for one species over the other. Otherwise, the deviations from a random expectation are small, and there is no real local preference of hosts by the parasite. We used the percentile bootstrap (Manly 1997) on the deviations from expectations for each host species separately. If values were lower than 0.5 (i.e., 0.5 not included in the 95% CI), then the parasite was usually closer to the expected species, and the results can be randomly obtained (because deviations from expectations are not that large). We used a randomization test (Manly 1997) to test for differences between the deviations from expectations, when the nearest host is *L. acervorum* or *L. muscorum*. Significant values should indicate on preference for one host species.

Results

On a coarser scale (among plots), we first tested for an association between host and slavemaker abundance. Slavemaker colony abundance correlated neither with *L. acervorum* (F_1,18_ = 0.01, P = 0.91, R^2^ < 0.001) nor with *L. muscorum* abundance (F_1,18_ = 0.58, P = 0.46, R^2^ < 0.001). On a finer scale and according to the null model, the distances of each slavemaker colony to one host species did not differ from the distances to the other host species (Randomization test: P = 0.71).
